# Altered Functional Connectivity of the Primary Visual Cortex in Patients With Iridocyclitis and Assessment of Its Predictive Value Using Machine Learning

**DOI:** 10.3389/fimmu.2021.660554

**Published:** 2021-05-07

**Authors:** Yan Tong, Xin Huang, Chen-Xing Qi, Yin Shen

**Affiliations:** ^1^ Eye Center, Renmin Hospital of Wuhan University, Wuhan, China; ^2^ Department of Ophthalmology, Jiangxi Provincial People’s Hospital, Nanchang, China; ^3^ Frontier Science Center for Immunology and Metabolism, Medical Research Institute, Wuhan University, Wuhan, China

**Keywords:** functional connectivity, iridocyclitis, primary visual area, functional magnetic resonance imaging, support vector machine, machine learning

## Abstract

**Purpose:**

To explore the intrinsic functional connectivity (FC) alteration of the primary visual cortex (V1) between individuals with iridocyclitis and healthy controls (HCs) by the resting-state functional magnetic resonance imaging (fMRI) technique, and to investigate whether FC findings be used to differentiate patients with iridocyclitis from HCs.

**Methods:**

Twenty-six patients with iridocyclitis and twenty-eight well-matched HCs were recruited in our study and underwent resting-state fMRI examinations. The fMRI data were analyzed by Statistical Parametric Mapping (SPM12), Data Processing and Analysis for Brain Imaging (DPABI), and Resting State fMRI Data Analysis Toolkit (REST) software. Differences in FC signal values of the V1 between the individuals with iridocyclitis and HCs were compared using independent two-sample t-tests. Significant differences in FC between two groups were chosen as classification features for distinguishing individuals with iridocyclitis from HCs using a support vector machine (SVM) classifier that involved machine learning. Classifier performance was evaluated using permutation test analysis.

**Results:**

Compared with HCs, patients with iridocyclitis displayed significantly increased FC between the left V1 and left cerebellum crus1, left cerebellum 10, bilateral inferior temporal gyrus, right hippocampus, and left superior occipital gyrus. Moreover, patients with iridocyclitis displayed significantly lower FC between the left V1 and both the bilateral calcarine and bilateral postcentral gyrus. Patients with iridocyclitis also exhibited significantly higher FC values between the right V1 and left cerebellum crus1, bilateral thalamus, and left middle temporal gyrus; while they displayed significantly lower FC between the right V1 and both the bilateral calcarine and bilateral postcentral gyrus (voxel-level *P*<0.01, Gaussian random field correction, cluster-level *P*<0.05). Our results showed that 63.46% of the participants were correctly classified using the leave-one-out cross-validation technique with an SVM classifier based on the FC of the left V1; and 67.31% of the participants were correctly classified based on the FC of the right V1 (*P*<0.001, non-parametric permutation test).

**Conclusion:**

Patients with iridocyclitis displayed significantly disturbed FC between the V1 and various brain regions, including vision-related, somatosensory, and cognition-related regions. The FC variability could distinguish patients with iridocyclitis from HCs with substantial accuracy. These findings may aid in identifying the potential neurological mechanisms of impaired visual function in individuals with iridocyclitis.

## Introduction

Iridocyclitis is the most common pattern of anterior uveitis among the general population and is characterized by the presence of an inflammatory phenotype within the anterior eye segment that involves the iris and ciliary body (1). The typical clinical features of patients with iridocyclitis include eye redness, eye pain, blurred vision, and photophobia. Aggravating features (e.g., secondary glaucoma, cystoid macular edema, corneal opacities and retinal vascular abnormalities) have also been frequently reported to cause vision loss and may ultimately result in irreversible blindness (2). Many affected patients have no evidence of an underlying disorder and their disease is considered idiopathic; however, iridocyclitis is also often associated with autoimmunity. Human leucocyte antigen (HLA-B27) is a critical risk factor for the onset of iridocyclitis; notably, approximately 40-70% of patients with anterior uveitis are HLA-B27 positive, whereas this proportion is only 8-10% of healthy individuals (3). Iridocyclitis is commonly related to HLA-B27-associated systemic immune-mediated diseases, including ankylosing spondylitis, inflammatory bowel disease and reactive arthritis (4, 5). Topical or systemic corticosteroids are often used; steroid sparing current treatment modalities include disease-modifying anti-rheumatic drugs, such as conventional or synthetic biological drugs (6).

Inflammatory and autoimmune pathologies in iridocyclitis affect both afferent and efferent visual functions. In-depth analysis of immunopathology has demonstrated that autoimmune responses induce or participate in the occurrence and development of iridocyclitis (7). Notably, patients with iridocyclitis can produce cellular or humoral immune responses to retinal tissue antigens, such as S-antigen, recoverin, and interphotoreceptor retinoid-binding protein (8). Additionally, the infiltration of the Th17 and Th1 cells is induced in experimental autoimmune uveitis, such that these cells can be detected in retinal tissues (9). The availability of optical coherence tomography has provided additional structural information concerning changes in the retinal morphology of patients with iridocyclitis. In particular, retinal nerve fiber layer, central foveal, and retinal thicknesses are affected during the active iridocyclitis, even in the presence of mild anterior inflammation (10, 11). Investigations thus far suggest that anterior chamber inflammation in patients with iridocyclitis might lead to various changes in the posterior segment, potentially also modifying brain activity. However, the mechanisms of visual pathway impairment and the patterns of spontaneous neuronal activity alterations in the primary visual cortex (V1) in individuals with iridocyclitis have not been fully elucidated.

Resting-state functional magnetic resonance imaging (rs-fMRI), a noninvasive neuroimaging method dependent on metabolism analysis and brain blood flow, is progressively enabling people to detect functional changes within specific cerebral region (12, 13). Among the rs-fMRI analysis techniques, functional connectivity (FC) technique – in which the temporal correlation is calculated in a low-frequency range (0.01-0.08 Hz) between the mean blood oxygen level-dependent time series of a pre-determined brain region and spatially separated brain regions – is the most extensively used method because of its straightforward nature, sensitive, and efficiently in providing insights into the patterns of neural interactions (14). The FC method has been successfully applied to explore spontaneous neural alterations in some vision-related diseases, such as retinal vein occlusion (15), strabismus (16), retinitis pigmentosa (17), and glaucoma (18). The V1 is located in the occipital lobe; this is the kernel of the visual cortex that involves in perceptual activities. It receives visual input from the lateral geniculate nucleus, conducts initial integration, then transmits that information to senior regions of the visual cortex for complex processing (19). To our knowledge, FC changes in the V1 in patients with iridocyclitis have not been reported.

Recently, MRI-related machine learning methods have provided a systematic approach for developing automatic, objective, and sophisticated classification networks for the analyses of high-dimensional data. These approaches are more sensitive to identify subtle differences in the brain structure than group-level statistics based on multivariate pattern (20, 21). The support vector machine (SVM) method is the most commonly used supervised machine learning algorithm for MRI classification that enables individual-level classification and detects biomarkers on the basis of neuroimaging data. In recent years, it has been used to distinguish patients from healthy controls (HCs) in several diseases [e.g., Parkinson’s disease (22), generalized anxiety disorder (23)] with high accuracy. To our best knowledge, no studies have used the SVM technique based on fMRI data to perform predictive investigations in patients with iridocyclitis.

Based on previous studies, we hypothesized that patients with iridocyclitis may have disturbances in spontaneous neuronal activity and visual function. Thus, this study was performed to assess alterations in FC signal values between the V1 and other cortical areas in individuals with iridocyclitis, with the aim of providing an in-depth analysis of the specific V1 connectivity in those patients. Moreover, machine learning techniques were applied to find out whether aberrant FC could serve to be a feature to distinguish patients with iridocyclitis from normal controls. The results may contribute to explore the potential neuronal mechanisms of impaired visual function in individuals with iridocyclitis and contribute to the diagnosis of iridocyclitis in clinical practice.

## Materials and Methods

### Participants

A total of 26 individuals with iridocyclitis (14 males and 12 females) were enrolled from the Eye Center, Renmin Hospital of Wuhan University. The inclusion criteria were as follow: (1) diagnosis of iridocyclitis by experienced ophthalmologist based on the Standardization of Uveitis Nomenclature working group classification (1); (2) no other ocular disease (glaucoma, high myopia, strabismus, optic atrophy etc.) (3) right handed preference; (4) no contraindications for MRI examination. The exclusion criteria were as follow: (1) history of eye surgery and ocular trauma; (2) presence of brain parenchyma or other systemic disease (e.g. hypertension, diabetes, and heart disease) (3) psychiatric disorders.

26 HCs (15 males and 11 females) comparably matched for sex, age, as well as education compared to the patient group participated in this research. The inclusion criteria were listed as below: (1) no eye disease (e.g. cataracts, optic neuritis, diabetic retinopathy, etc.); (2) visual acuity ≥ 1.0 in both eyes; (3) right handed preference; (4) no psychiatric diseases; (5) capable of undergoing MRI examination.

The study followed the tenets of the in the Declaration of Helsinki, and was formally approved by the institutional review board of Eye Center, Renmin Hospital of Wuhan University. Each participant was provided signed informed consent to participate in our study.

### MRI Parameters

A 3.0-Tesla magnetic resonance scanner (Discovery MR 750W system; Bio-Sciences Corporation) with an eight-channel head coil was used to collect fMRI images. All participates were asked to remain awake, lay still, and keep their eyes closed until the examination was completed. A three-dimensional spoiled gradient-recalled echo sequence was conducted to collect the anatomical T1-weighted images. The whole brain fMRI data was recorded by applying gradient-recalled echo-planar imaging sequence. More detailed parameter setting was displayed in **Table 1**. In total, 240 volumes of fMRI data were obtained for the next pre-processing analysis.

**Table 1:** Parameters setting of the MRI scanning.

Data acquisition	Brain volume sequence	Echo-planar imaging sequence
Repetition time	1,900 ms	2,000 ms
Echo time	2.26 ms	30 ms
Gap	0 mm	1.2 mm
Field of view	240 x 240 mm^2^	240 x 240 mm^2^
Acquisition matrix	256 x 256	64 x 64
Flip angle	12°	90°

### fMRI Data Pre-Processing Analysis

The data were preprocessed using the Statistical Parametric Mapping (SPM12) and Data Processing and Analysis for Brain Imaging (DPABI) toolboxes running on Matlab 2013b (24). The preprocessing applied in the research were as below: (1) the fMRI data were transformed from DICOM format to NIFTI format; (2) the first 10 volumes of the functional images were excluded to reach magnetization equilibrium; (3) the rest 230 functional volumes were corrected with respect to slice timing, and head motion correction, then realigned (fMRI data from participates with greater than 1.5 mm displacement in any direction or more than 1.5° angular motion were discarded). Besides, we compared head motion parameters between the two groups and observed no significant differences (**Table 2**). (4) individual T1-weighted images were co-registered to the average functional data and the processed data were all segmented through the Diffeomorphic Anatomical Registration Through Exponentiated Lie Algebra algorithm to enhance spatial precision during the normalization of functional data (25). Normalized fMRI data were re-sliced with a resolution of 3 x 3 x 3 mm^3^; (5) data were smoothed with a 6 x6 x 6 mm^3^ full-width at half-maximum Gaussian Kernel; (6) removal of data with linear trends; linear regression step was conducted to control for some covariates including six head motion parameters, white matter signal, and cerebrospinal fluid signal; (7) temporal band-pass filtering was performed (0.01-0.08 Hz).

**Table 2:** Clinical characteristics for patients with iridocyclitis and HCs.

	Iridocyclitis group	HC group	*T*-values	*P*-values
Sex (male/female)	14/12	15/11	N/A	0.780
Age (years)	45.15 ± 14.95	45.30 ± 13.87	-0.038	0.970
Education (years)	11.04 ± 3.94	11.52 ± 3.48	-0.472	0.639
BCVA-OD	0.44 ± 0.27	1.16 ± 0.16	-11.474	<0.001*
BCVA-OS	0.43 ± 0.37	1.19 ± 0.16	-9.352	<0.001*
Duration of iridocyclitis (days)	4.43 ± 2.89	N/A	N/A	N/A
Handedness	26 R	26 R	N/A	N/A
Head motion (mm)	0.102 ± 0.941	0.086 ± 0.055	0.765	0.448

Chi-square test for sex. Independent t-test was used for other normally distributed continuous data. Data are presented as mean ± standard deviation.

HC, healthy control; BCVA, best-corrected visual acuity; OD, oculus dexter; OS, oculus sinister; N/A, not applicable; R, right. The symbol * denotes p < 0.05.

### Definition of Region of Interest

In the fMRI data analysis for each participant, regions of interest were defined using the DPABI toolkit. Each side of the V1 was selected, which generally resulted in a region of interest containing Brodmann’s area 17. The center of V1 was selected to be the seed point and the MNI coordinates of the bilateral V1 were (left: -8, -76, 10) and (right: 8, -76, 10), in accordance with previous methods (26). The diameter of the sphere region of interest was 6 mm. Pearson correlation coefficients were computed between the average time series of the regions of interest and the time series for other cerebrum regions. Moreover, Fisher’ r-to-z-transformation was used to acquire an approximate normal distribution and help to reduce the impacts of individual variations for group statistical comparisons.

### Statistical Analysis

SPSS 19.0 software was adopted in the study to analyze the cumulative clinical and demographic data. Independent two-samples *t*-test were used to evaluate continuous variables, while the chi-square test was used to assess proportions (*P*<0.05 indicates statistically significant). One-sample t-test was conducted to evaluate intragroup patterns of z-value FC; two-samples t-test were performed to compare differences in z-value FC between two groups using SPM12 software (voxel-level: *P*< 0.01, Gaussian random field (GRF) correction), cluster-level: *P*<0.05). Furthermore, Pearson correlation coefficients were utilized to evaluate the relationships between the average FC signal values in distinct cerebral areas and the clinical features in patients with iridocyclitis (*P*<0.05 indicates statistically significant).

### Support Vector Machine Analysis

The SVM algorithm based on the Pattern Recognition for Neuroimaging Toolbox (PRoNTo) software (http://www.mlnl.cs.ucl.ac.uk/pronto/prtsoftware.html) was used to conduct diagnostic prediction in patients with iridocyclitis. The PRoNTo software has five main analysis modules: (i) dataset specification; (ii) feature set selection; (iii) model specification; (iv) model estimation; (v) weights computation (27). The SVM approach in PRoNTo utilizes LIBSVM for Matlab, which is an implementation of a linear-kernel SVM for binary classification (28).

In the present study, individual participant’s FC maps served as inputs for the machine learning algorithm. A feature set was prepared on whole brain FC maps and the number of features is the total number of edges $\left(C_{90}^{2}for\;AAL90\;brainatlas\right)$. An important feature of SVM is that it does not need the training and test data to be explicitly represented and instead can be trained solely using a kernel, which is a matrix of pairwise similarities between data points constructed from the original features. Thus, the computational complexity of SVM is governed by the number of samples, rather than the number of features, which is advantageous in high-dimensional settings. Above procedures were automatically processed in PRoNTo’s “Prepare feature set” programs.

During the training process, one way to optimize the classifier and estimate the accuracy of the algorithm is by using the cross-validation method. The leave-one-out cross-validation (LOOCV) technique was adopted to perform SVM classifier validation, where the feature selection was performed each time on the training partition of the data. The number of samples in our study was assumed to be *n*. In each LOOCV fold, FC data from *n*-1 samples were selected as the training dataset to train the classification model; FC data from the remaining sample were regarded as a test dataset to test the ability of the classifier to classify new cases reliably. This procedure was repeated *n* times. For classification, two classes were defined (patients group and HCs group) and processed using a soft-margin hyper-parameter approach. The soft-margin parameters take the values 0.01, 0.1, 1, 10, 100 and 1000 in the SVM classifier in the current version of PRoNTo, which make the model obtain the maximum interval hyperplane with the minimum classification error. There is an inner-loop in the PRoNTo software to automatically find the optimal soft-margin parameter. The PRoNTo calculated the total error rate of model classification under six soft-margin parameters, and then selected the soft-margin parameter with the lowest total error rate as the final parameter for each cycle of the cross-validation. Above steps were automatically processed in the “Specify model” program in PRoNTo.

The total accuracy, specificity, sensitivity, and area under the receiver operating characteristic curve (AUC) were determined to assess the classification performance of the machine learning model. The permutation test was applied to assess the statistical significance of the total accuracy of this classification (29). The permutation test was repeated 1,000 times. During each repeat, the SVM model reallocated labels of iridocyclitis and normal controls randomly to the training participants and repeated the whole classification process. The p-value was acquired after all permutations had been completed.

To improve the interpretability of the multivariate pattern recognition results, we calculated the images summarizing the weights of each region of interest (ROI) defined by the Anatomical Automatic Labeling (AAL) atlas, which includes 116 cortical and subcortical anatomical structures (27). The weight map displays the contribution of each voxel in the image for the linear predictive function. Since each cross-validation fold yields a different weight vector, the final weight map is the average map across the folds divided by its Euclidean norm. Here, for each brain region defined by the AAL template, the normalized weight is calculated as the mean of absolute values of all voxel weights within the ROI. Region contributions can be ranked in descending order, and a sorted list of regions is obtained according to their contribution to the classification model based on the percentage of the total normalized average weights (30, 31).

## Results

### Demographics and Clinical Data Comparison

**Figure 1:**
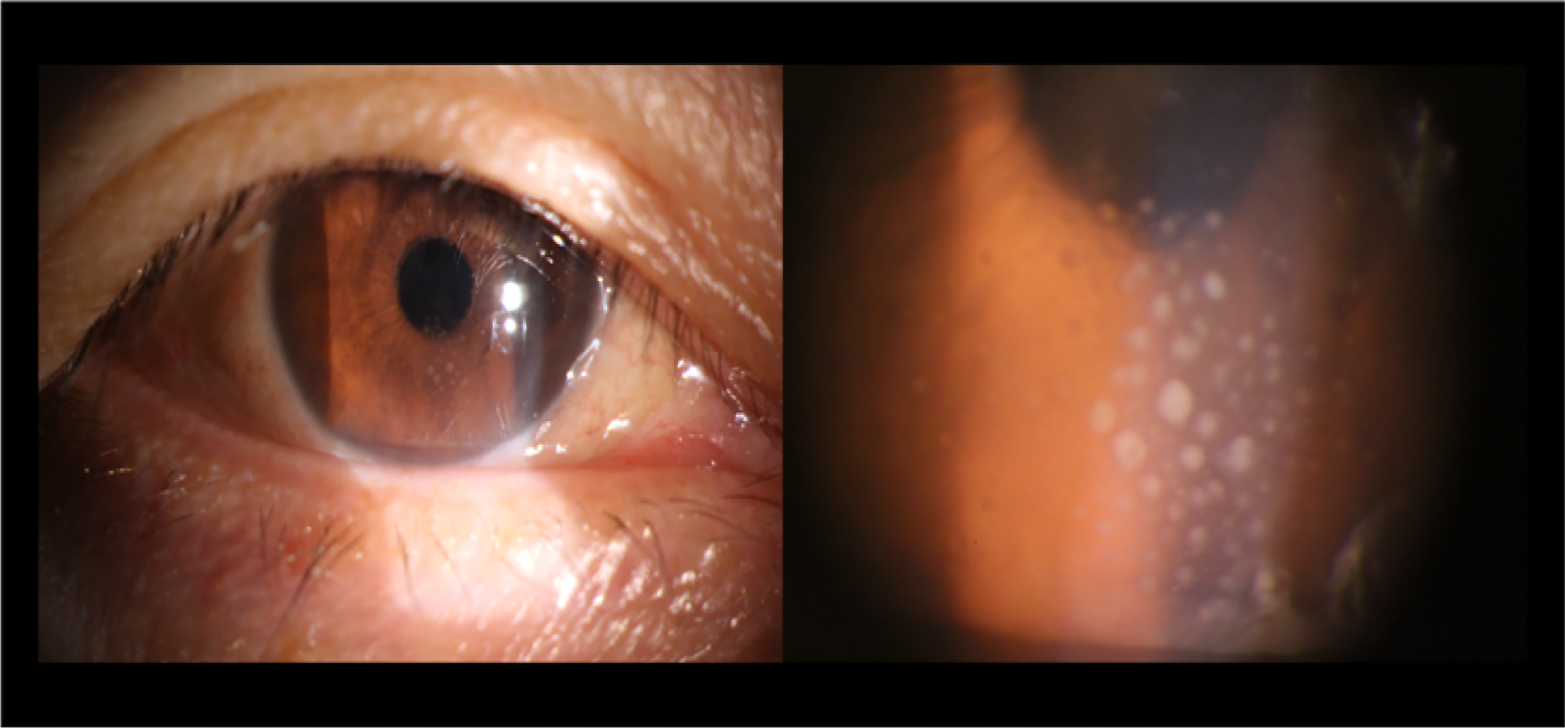
Typical anterior segment photographs of iridocyclitis.

The results of demographic as well as clinical data are displayed in **Table 2**. There were no markedly differences in gender (*P*=0.780), age (*P*=0.97), or education (*P*=0.871) between the iridocyclitis patients group and HCs group. By contrast, the statistically differences were observed in the best-corrected visual acuity between the patient group and normal control group (*P*<0.001). The typical anterior segment photographs were shown in **Figure 1**.

### Comparisons of FC Between Patients With Iridocyclitis and HCs

**Figure 2:**
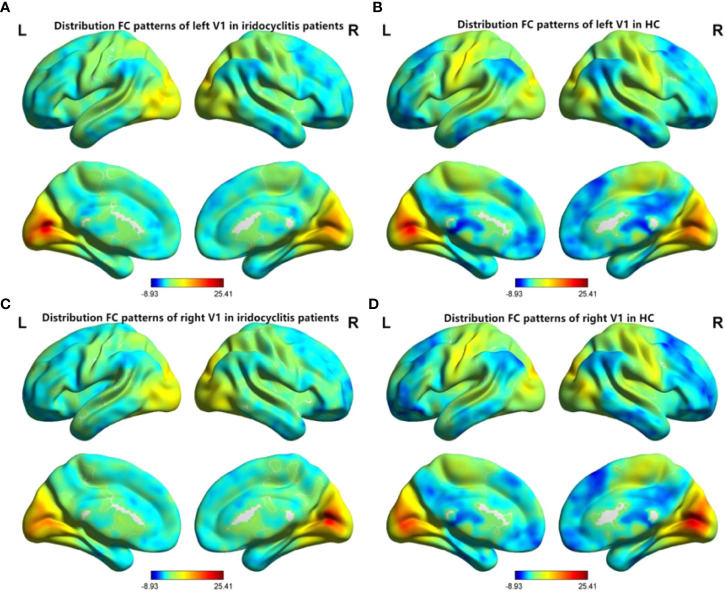
Spatial distributions of FC patterns of the bilateral V1 of the patients with iridocyclitis and HCs. **(A)** Distribution average zFC values of left V1 in patients with iridocyclitis; **(B)** Distribution average zFC values of left V1 in HCs; **(C)** Distribution average zFC values of right V1 in patients with iridocyclitis; **(D)** Distribution average zFC values of right V1 in HCs. zFC, z-value functional connectivity; HC, healthy control.

**Figure 3:**
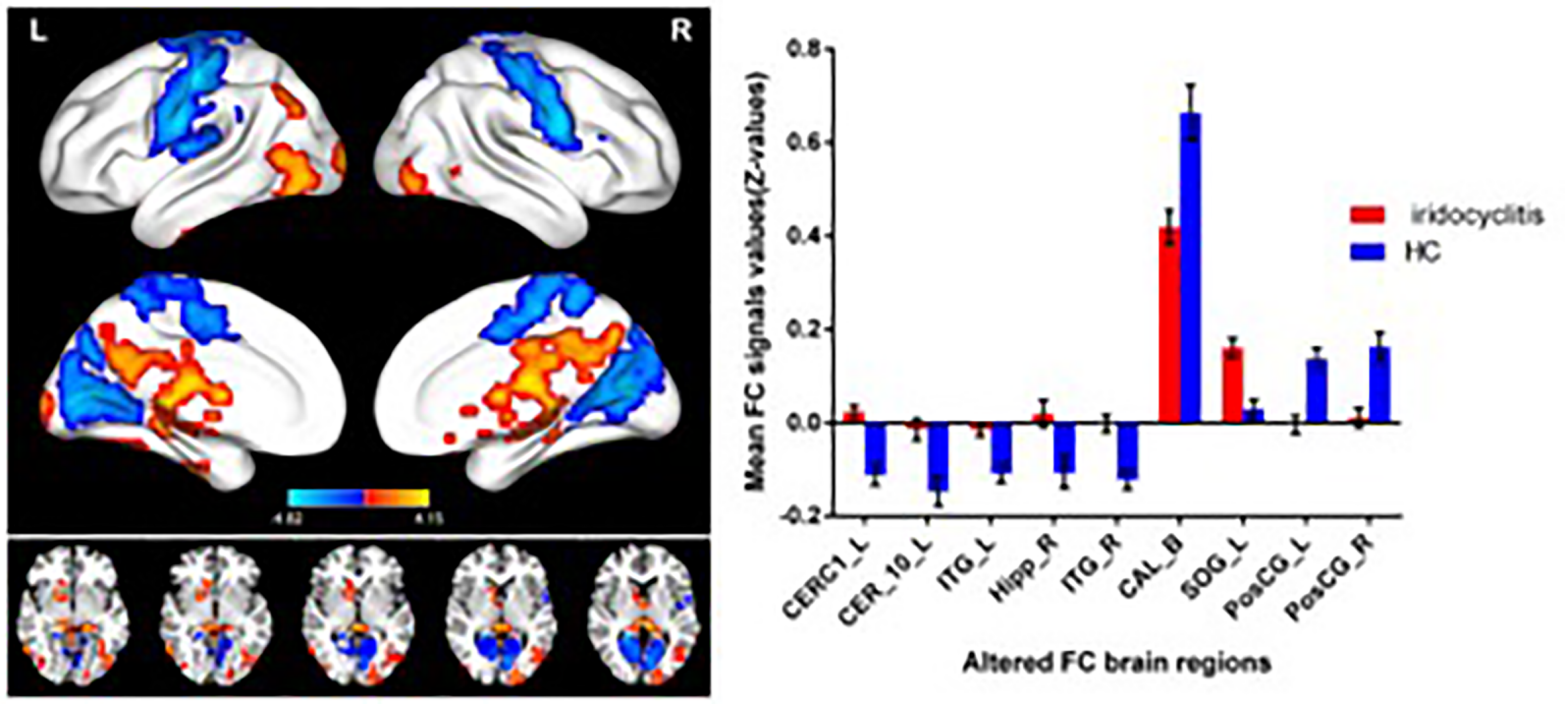
Brain areas with statistically significant differences between two groups in FC of the left V1. Significantly FC differences were shown between the left V1 and CERC1_L, CER_10_L, ITG_L, Hipp_R, ITG_R, CAL_B, SOG_L, PosCG_L and PosCG_R. (voxel-level *P*<0.01, GRF correction, cluster-level *P*<0.05). FC, functional connectivity; CERC1_L, left cerebellum crus1; CER_10_L, left cerebellum 10; ITG_L, left inferior temporal gyrus; Hipp_R, right hippocampus; ITG_R, right inferior temporal gyrus; CAL_B, bilateral calcarine; SOG_L, left superior occipital gyrus; PosCG_L, left postcentral gyrus; PosCG_R, right postcentral gyrus.

**Figure 4:**
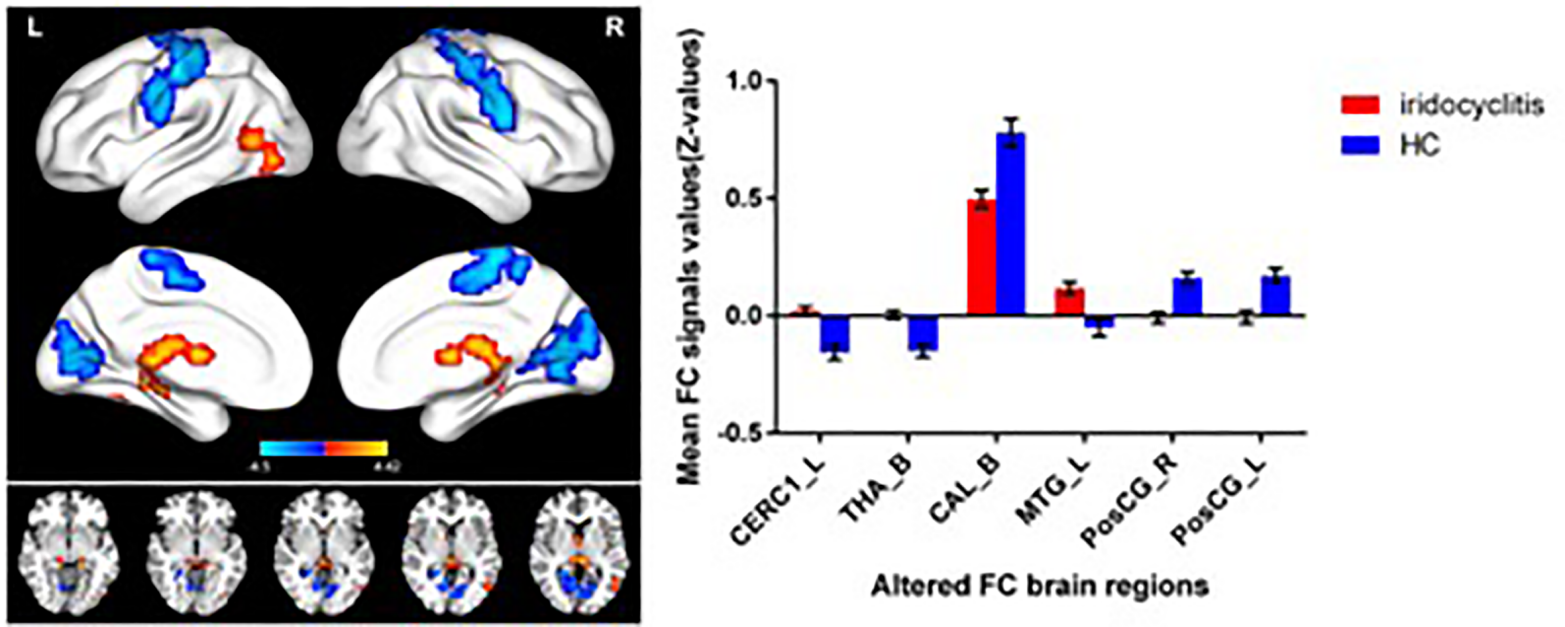
Brain areas with statistically significant differences between two groups in FC of the right V1. Significantly FC differences were shown between the right V1 and CERC1_L, THA_B, MTG_L, CAL_B, PosCG_L and PosCG_R (voxel-level *P*<0.01, GRF correction, cluster-level *P*<0.05). FC, functional connectivity; CERC1_L, left Cerebellum Crus1; THA_B, bilateral thalamus; CAL_B, bilateral calcarine; MTG_L, left middle temporal gyrus; PosCG_L, left postcentral gyrus; PosCG_R, right postcentral gyrus.

**Figure 2** shows the spatial distributions of average zFC values of the bilateral V1 in patients with iridocyclitis and HCs. Compared with HCs, individuals with iridocyclitis displayed markedly higher FC between the left V1 and left cerebellum crus1, left cerebellum 10, bilateral inferior temporal cortex, right hippocampus, and left superior occipital gyrus; they showed significantly lower FC between the left V1 and both the bilateral calcarine and bilateral postcentral gyrus (**Figure 3** and **Table 3**). Furthermore, patients with iridocyclitis displayed significantly increased FC signal values between the right V1 and left cerebellum crus1, bilateral thalamus, and left middle temporal gyrus; they demonstrated significantly lower FC signal values between the right V1 and both the bilateral calcarine and bilateral postcentral gyrus (**Figure 4** and **Table 4**) (voxel-level *P*<0.01, GRF correction, cluster-level *P*<0.05).

**Table 3:** Significant differences in the left V1 between two groups.

Condition	Brain regions	BA	Peak T-scores	MNI coordinates			Cluster size (voxels)
				x	y	z	
iridocyclitis >HC	CerC1_L	**-**	4.1486	-6	-72	-51	4014
iridocyclitis >HC	CER_10_L	–	3.5071	-21	-39	-42	49
iridocyclitis >HC	ITG_L	20	3.281	-48	-18	-33	79
iridocyclitis >HC	Hipp_R	–	2.8445	21	-12	-18	42
iridocyclitis >HC	ITG_R	19	3.3436	60	-63	-3	58
iridocyclitis >HC	SOG_L	17	3.2622	-18	-87	6	286
iridocyclitis <HC	CAL_B	18	-4.8233	21	-48	9	1440
iridocyclitis <HC	PosCG_L	6	-4.6021	-51	-18	48	1719
iridocyclitis <HC	PosCG_R	6	-4.4712	66	0	21	901

The statistical threshold was set at the voxel level with p<0.01 for multiple comparisons using Gaussian random-field theory (two-tailed, voxel-level P<0.01, GRF correction, cluster-level P < 0.05).

CERC1_L, left cerebellum crus1; CER_10_L, left cerebellum 10; ITG_L, left inferior temporal gyrus; Hipp_R, right hippocampus; ITG_R, right inferior temporal gyrus; CAL_B, bilateral calcarine; SOG_L, left superior occipital gyrus; PosCG_L, left postcentral gyrus; PosCG_R, right postcentral gyrus; BA, Brodmann’s area.

**Table 4:** Significant differences in the right V1 between two groups.

Condition	Brain regions	BA	Peak T-scores	MNI coordinates			Cluster size (voxels)
				x	y	z	
iridocyclitis >HC	CERC1_L	**-**	4.094	-21	-78	-27	889
iridocyclitis >HC	THA_B	–	4.4152	-6	-30	6	339
Iridocyclitis<HC	CAL_B	18	-4.4069	21	-48	9	851
iridocyclitis >HC	MTG_L	–	3.6074	-51	-63	6	55
iridocyclitis <HC	PosCG_R	6	-4.0193	57	-9	36	994
iridocyclitis <HC	PosCG_L	3	-4.5003	-51	-18	48	580

The statistical threshold was set at the voxel level with p<0.01 for multiple comparisons using Gaussian random-field theory (two-tailed, voxel-level P<0.01, GRF correction, cluster-level P < 0.05).

CERC1_L, left Cerebellum Crus1; THA_B, bilateral thalamus; CAL_B, bilateral calcarine; MTG_L, left middle temporal gyrus; PosCG_L, left postcentral gyrus; PosCG_R, right postcentral gyrus. BA, Brodmann’s area.

### SVM Classification Results

**Figure 5:**
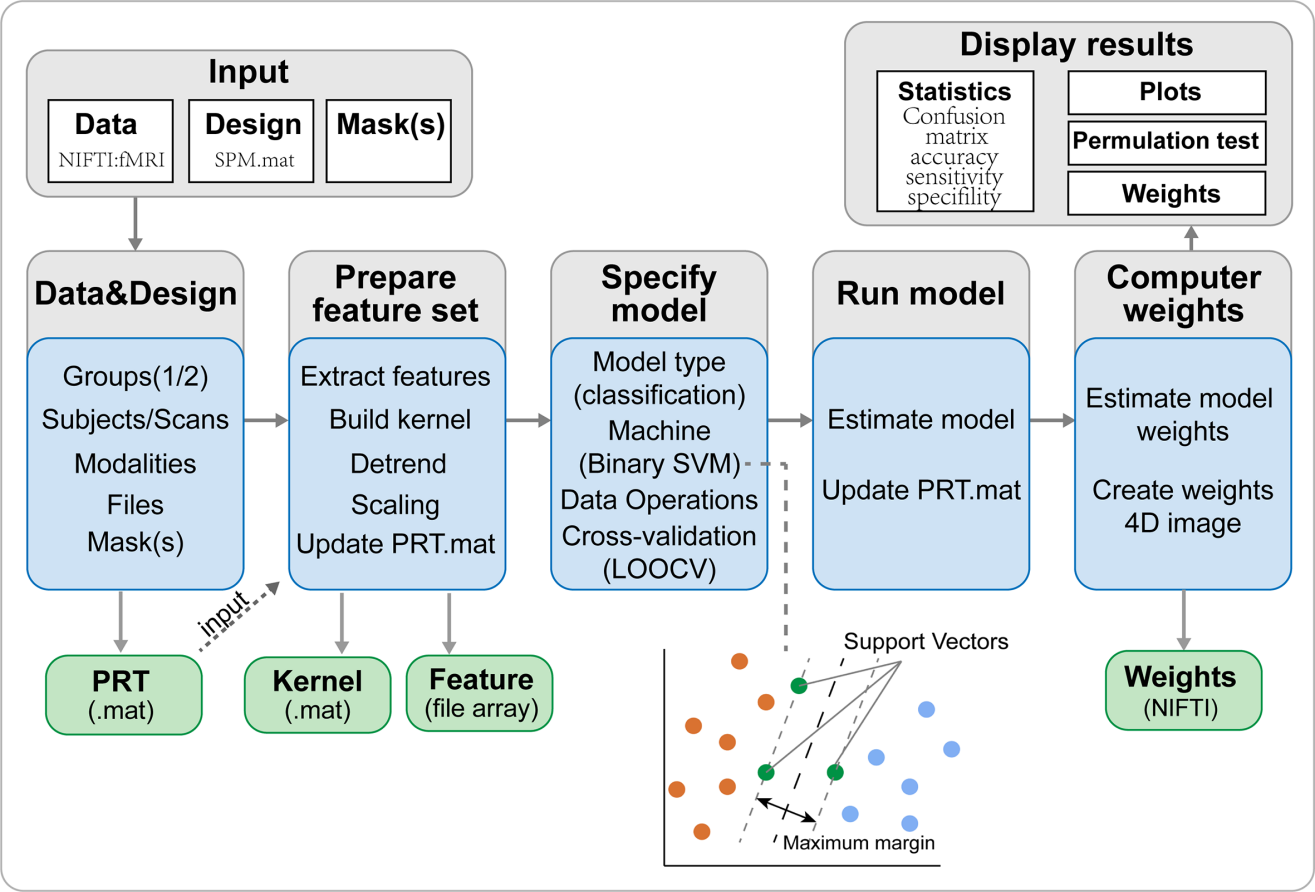
The framework of PRoNTo. The PRoNTo software has five main analysis modules: (i) dataset specification; (ii) feature set selection; (iii) model specification; (iv) model estimation; (v) weights computation. The PRT.mat is the output data structure from PRoNTo. SVM, support vector machine; LOOCV, SVM, support vector machine.

**Figure 6:**
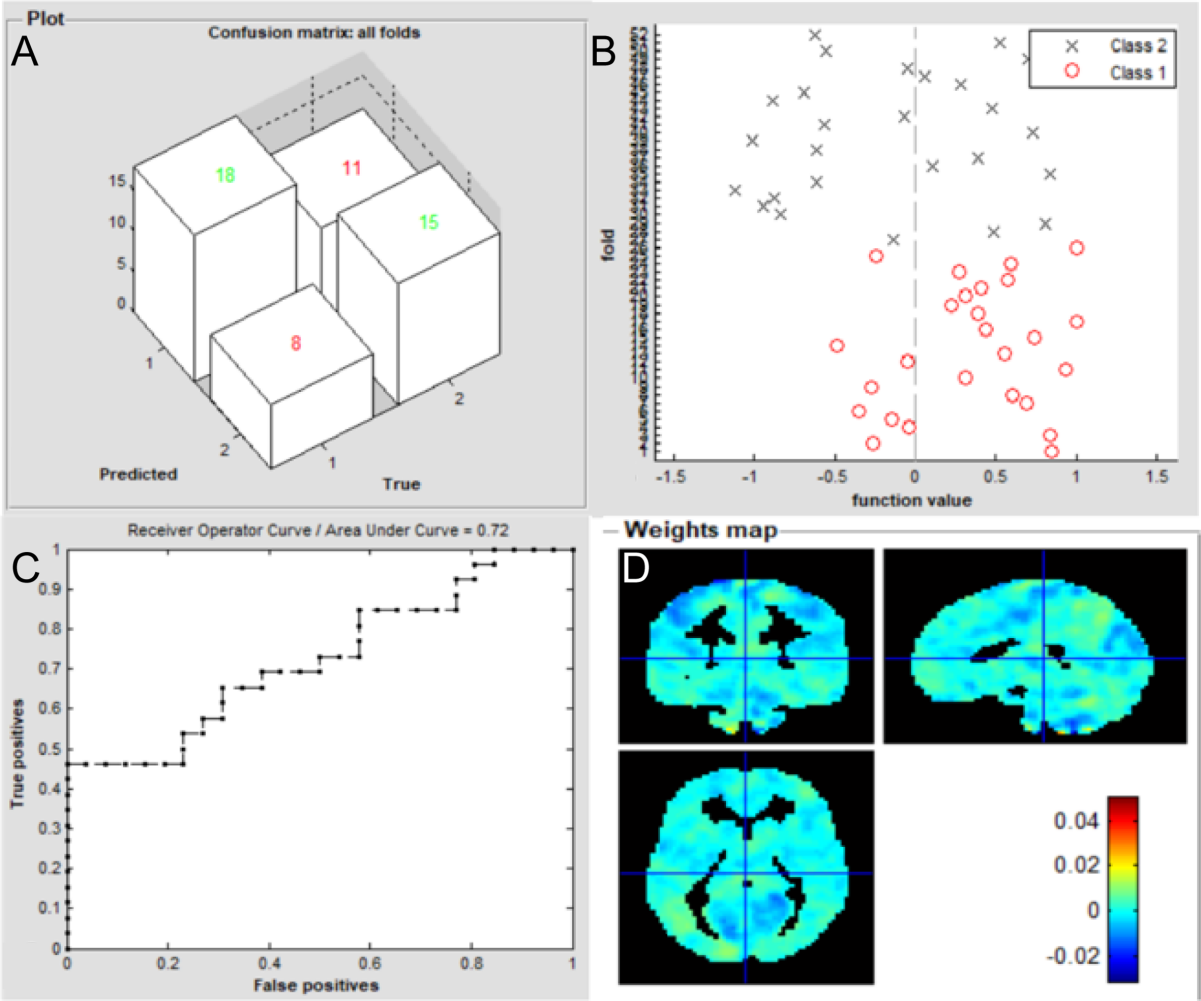
Classification results using machine learning analysis based on FC of the left V1. **(A)** three-dimensional confusion matrices from machine learning analysis; **(B)** function values of two groups (class 1: patient group; class 2: HC group); **(C)** The ROC curve of the classifier, and the AUC was 0.72; **(D)** Weight maps for SVM models. The weight in each voxel corresponding to its contribution to the model’s prediction.

**Figure 7:**
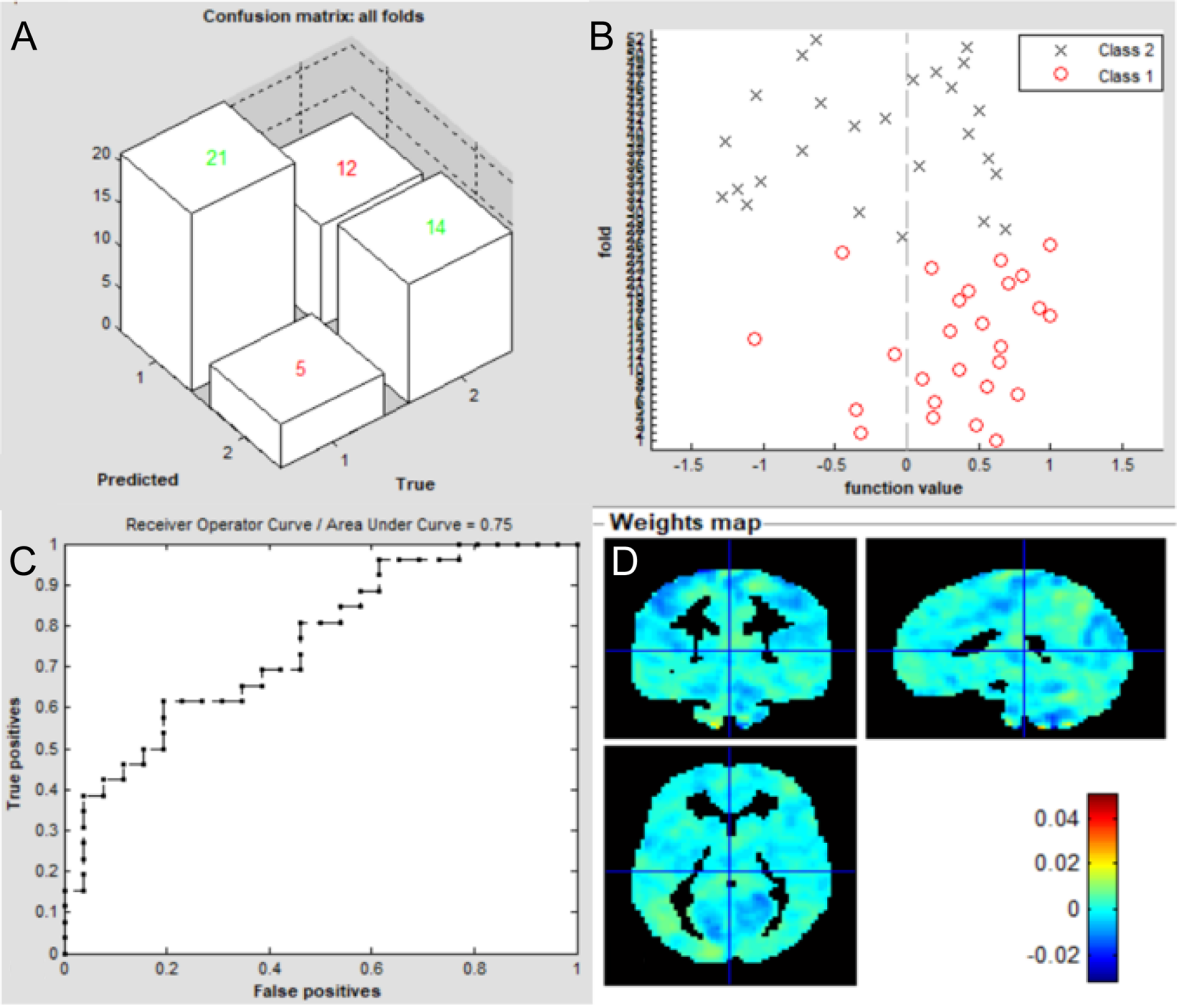
Classification results using machine learning analysis based on FC of the right V1. **(A)** three-dimensional confusion matrices from machine learning analysis; **(B)** function values of two groups (class 1: patient group; class 2: HC group); **(C)** The ROC curve of the classifier, and the AUC was 0.75; **(D)** Weight maps for SVM models. The weight in each voxel corresponding to its contribution to the model’s prediction.

The overall flowchart of the PRoNTo is shown in **Figure 5**. Classification results based on FC of the left V1 are displayed in **Figure 6**. The SVM classification model applying the LOOCV method achieved a total accuracy of 63.46%, sensitivity of 69.23%, and specificity of 65.22%. The AUC of the classification model was 0.72. Additionally, classification results based on FC of the right V1 are displayed in **Figure 7**. The SVM classification model achieved a total accuracy of 67.31%, sensitivity of 80.77%, and specificity of 53.85%. The AUC of the classification model was 0.75 (*P*<0.001, non-parametric permutation test).

### Regions Contributed Most for Classification

For FC of the left V1, the most informative regions for classification between patients of iridocyclitis and HC included the vermis_9; left inferior occipital gyrus; right inferior occipital gyrus; left postcentral gyrus and cerebelum_6_L. While for FC of the right V1, the most informative regions for classification included the right paracentral lobule; cerebelum_10_L; cerebelum_6_L; right superior parietal gyrus and right inferior occipital gyrus. Detailed results could be obtained in **Table 5**.

**Table 5:** Brain areas contributed most for classification between iridocyclitis and HC groups for the FC feature.

Brain regions	Discriminative weight (%)	ROI index	Cluster Size
**FC of the left V1**			
Vermis_9	1.862	115	44
Left inferior occipital gyrus	1.678	53	309
Right inferior occipital gyrus	1.592	54	275
Left postcentral gyrus	1.577	57	1093
Cerebelum_6_L	1.442	99	502
**FC of the right V1**			
Right paracentral lobule	2.978	70	220
Cerebelum_10_L	2.831	107	44
Cerebelum_6_L	1.624	99	502
Right superior parietal gyrus	1.612	60	601
Right inferior occipital gyrus	1.389	54	275

HC, healthy controls; FC, functional connectivity; ROI, region of interest; L, left.

## Discussion

**Figure 8:**
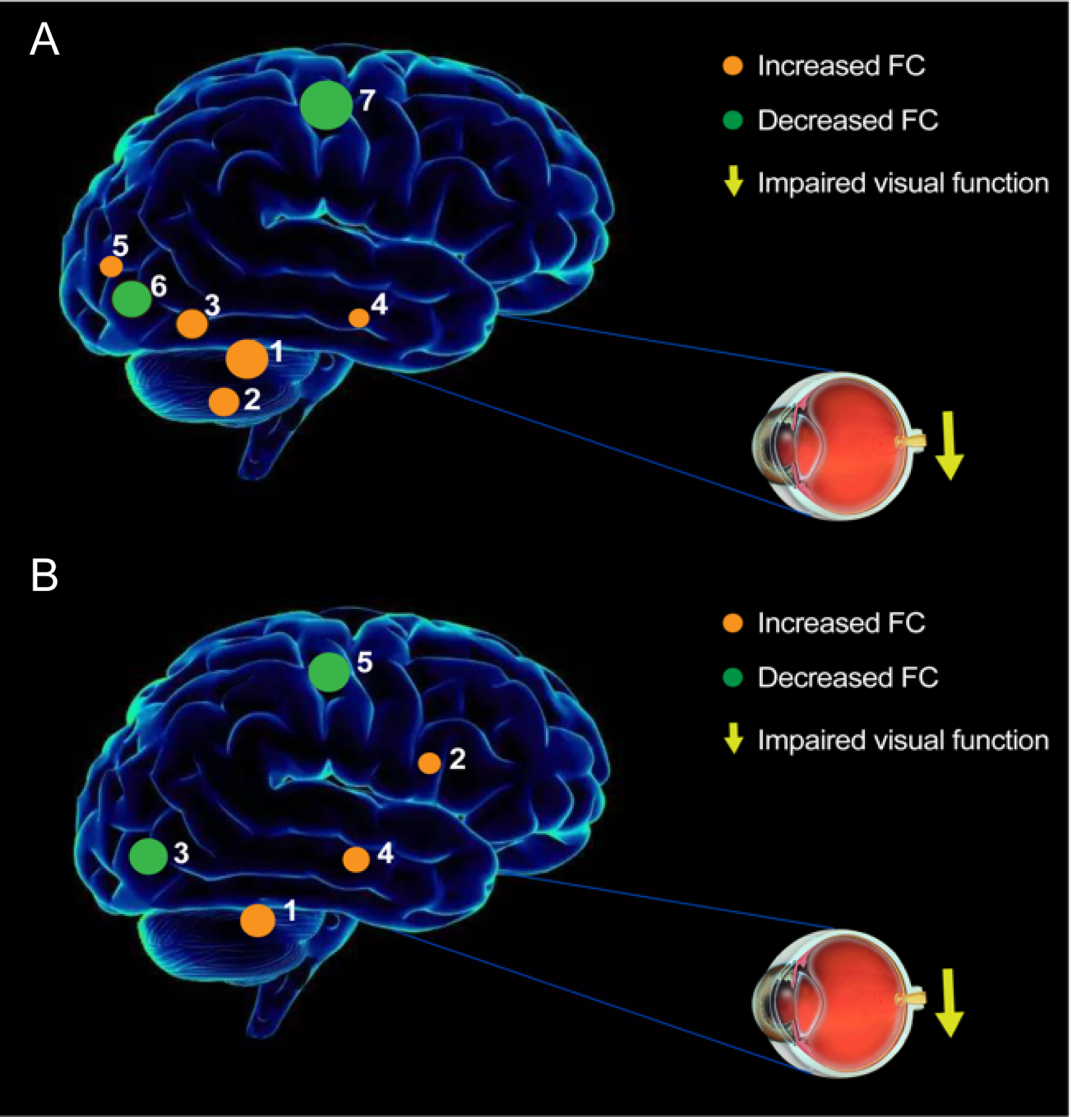
The FC pattern results of left V1 **(A)** and right V1 **(B)** in the iridocyclitis group. **(A)** Compared with HCs, the FC of left V1 in patients with iridocyclitis were increased to various extents: [1] left cerebellum crus1 (t=4.1486); [2] left cerebellum 10 (t=3.5071); [3] left inferior temporal gyrus (t=3.281); [3] right inferior temporal gyrus (t=3.3436); [4] right hippocampus (t=2.8445); [5] left superior occipital gyrus (t=3.2622), and the following areas decreased: [6] bilateral calcarine (t=-4.8233); [7] left postcentral gyrus (t=-4.6021); [7] right postcentral gyrus (t=-4.4712). **(B)** Compared with HCs, the FC of right V1 in patients with iridocyclitis were increased to various extents: [1] left cerebellum crus1 (t=4.094); [2] bilateral thalamus (t=4.4152); [4] left middle temporal gyrus (t=3.6074), and the following areas decreased: [3] bilateral calcarine (t=-4.4069); [5] left postcentral gyrus (t=-4.0193); [5] right postcentral gyrus (t=-4.5003).

The seed-based FC technique applied in our study offers a reliable and effective tool to evaluate the correlation coefficients of blood oxygen level-dependent signal time series between distinct brain areas. This approach has been widely applied in patients with ophthalmic diseases and has shown distinct FC disturbances between the V1 and other brain regions (**Table 6**). To our knowledge, this study was the first to explore FC between the V1 and other brain areas in individuals with iridocyclitis, which may provide a more exhaustive view on the specific connectivity of the V1 in iridocyclitis. In this study, we observed that compared to HCs, patients with iridocyclitis displayed significantly increased FC between the left V1 and left cerebellum crus1, left cerebellum 10, bilateral inferior temporal cortex, right hippocampus and left superior occipital gyrus; and significantly lower FC between the left V1 and bilateral calcarine, bilateral postcentral gyrus. Meanwhile, patients with iridocyclitis displayed significantly increased FC between the right V1 and left cerebellum crus1, bilateral thalamus, left middle temporal gyrus; and significantly lower FC between the right V1 and bilateral calcarine, bilateral postcentral gyrus **(** **Figure 8**, voxel-level *P*<0.01, GRF correction, cluster-level *P*<0.05**)**. Compared with other fMRI indices (e.g., regional homogeneity) which mainly focus on the local spontaneous brain activity alterations, the FC method is an effective tool to explore the neural activity connections between separated brain regions. Additionally, FC is based on a region of interest, which enables researchers to target a prior brain area before calculating its temporal coherence with other brain regions.

**Table 6:** FC technique used in ophthalmological diseases.

Author	Disease	Year	Brain areas	
			PG>HC	PG<HC
Su et al. (15)	Retinal vein occlusion	2020	RMFG; RSFG	RCUN
Wen et al. (17)	Retinitis pigmentosa	2018	RSC; LHipp	CUN/LGG/CAL; LPreG/PostG
Yu et al. (32)	Proliferative diabetic retinopathy	2017	RMFG; RSFG	CUN/CAL/PreCUN
Zhu et al. (33)	Comitant exotropia	2018	N/A	RIPL/PostG; RMOG; LLG/CPL LPreG/PostG;
Zhu et al. (34)	Corneal ulcer	2019	N/A	RMFG; RSFG; LIPL
Li et al. (18)	Primary angle-closure glaucoma	2017	LFOIBG; RIPL; LTP; RIBG	RCUN; CAL; RLG

FC, functional connectivity; PG: patient group; HC healthy control; CUN/LGG/CAL, cuneus/lingual gyrus/calcarine; RSC, right superior colliculus; LHipp, left hippocampus; RCUN, right cuneus; RSFG, right superior frontal gyrus; CUN/CAL/PreCUN, cuneus/calcarine/precuneus; RIPL/PostG, right inferior parietal lobule/postcentral gyrus; RMFG, right middle frontal gyrus; RMOG, right middle occipital gyrus; LLG/CPL, left lingual gyrus/cerebellum posterior lobe; RLG, right lingual gyrus; LIPL, left inferior parietal lobule; LPreG/PostG, left precentral gyrus/postcentral gyrus; LFOIBG, left frontal opercula-insula-basal ganglia region; LTP, left temporal-parietal region; RIBG, right insula-basal ganglia region; N/A, not applicable.

A major FC change was observed between the bilateral V1 and calcarine in patients with iridocyclitis. Anatomically, the calcarine (Brodmann’s area 18) is a constituent part of the occipital lobe, which takes a critical part in the vision network and is involved in visual imagery, visual color, and visual perception (35). Previous neurophysiological studies showed that visual signals were transmitted to the visual cortex through the visual pathway, which consists of dorsal and ventral streams (36). Because of this crucial function in the visual pathway, lesions of the calcarine are reportedly associated with the perceptions of phosphenes and visual field defects (37). Morphological research has implied that retinal nerve fiber layer, central foveal, and retinal thicknesses are affected during the progression of iridocyclitis, which may lead to reduced retinal input and diminished light stimulation at the visual center. Previous researches discovered that patients with iridocyclitis showed vision loss and reduced visual acuity (38). Furthermore, we revealed that patients with iridocyclitis exhibited significant reduction of regional homogeneity in bilateral calcarine and other portions of vison-related areas such as the right inferior occipital gyrus and left superior occipital gyrus in our previous study (39). Consistent with previous findings, our study discovered that individuals with iridocyclitis displayed markedly reduced FC between the bilateral V1 and calcarine. Accordingly, we hypothesized that the lower FC between the V1 and higher order visual cortex contributed to a range of vision loss in patients with iridocyclitis.

We also found significantly lower FC values between the V1 and bilateral postcentral gyrus in individuals with iridocyclitis. The postcentral gyrus is the anatomical area of the primary somatosensory cortex (S1), which participates in various types of sensory perception, including pain and touch (40, 41). The spontaneous neural activity of the visual cortex and S1 exhibit a robust correlation in normal-sighted individuals at rest, which facilitates the combined processing of somatosensory and spatial visual information (42, 43). Notably, we observed that patients with iridocyclitis showed abnormal local synchronization in the right postcentral gyrus during rest state using the regional homogeneity method, which might also contribute to the decrease FC (39). Therefore, we speculate that vision impairment could contribute to declining FC between the V1 and postcentral gyrus in individuals with iridocyclitis. Moreover, decreased visual input may reflect cross-modal plasticity in the V1. Previous fMRI studies have demonstrated that tactile stimuli activate the visual cortex *via* the somatosensory cortex in individuals with vision impairment (e.g., patients with late blind) (44). Thus, we concluded that the visual cortex participates in visual information transmission to the somatosensory processing pathway; moreover, vision loss and visual cortex representations of sensory modalities might lead to the lower FC between the V1 and S1 in patients with iridocyclitis. Furthermore, various pain-related diseases are reportedly related to S1 dysfunction (45, 46). Patients with iridocyclitis often exhibit typical clinical features of eye pain, consistent with our results.

The cerebellum, which functionally interacts with the frontal eye fields, participates in visuomotor coordination, higher cognitive function, and memory (47, 48). Iridocyclitis is considered the foremost clinical characteristic of ankylosing spondylitis; Li et al. discovered that individuals with ankylosing spondylitis exhibited greater activation in the cerebellum on fMRI analyses (49). In addition, the results of our previous study demonstrated that patients with iridocyclitis had enhanced regional homogeneity values in regions of the right cerebellum (39). Similarly, we found higher FC between the V1 and cerebellum (left cerebellum crus 1 and left cerebellum 10) in the present study. Therefore, patients with iridocyclitis may experience dysfunctional visuomotor function. Additionally, it was implied that neural hyperactivity between the cerebellum and V1 could be a compensatory reaction to vision loss in patients with iridocyclitis.

Patients with iridocyclitis displayed increased FC signal values between the V1 and inferior temporal gyrus (ITG). Importantly, the ITG is located in the protuberance or convolution of the cerebral hemisphere temporal lobe, which is situated below the middle temporal neocortex and the ITG area extends to the inferior sulcus. This area is involved in visual information processing (e.g., the classification of visual shape), object memory, and emotional regulation (50, 51). It is also associated with facial recognition and visual perception (52). Abnormalities in the ITG have been observed in multiple diseases, such as optic neuritis (53), Alzheimer’s disease (54), and blindness (26). Thus, the abnormal FC between the V1 and ITG in our study may result in difficulty with item identification and visual memory, as well as visual selectivity. Notably, Stouter et al. discovered that somatic pain disorder was related to enhanced activation of the temporal lobe (55), which is consistent with our finding that FC signals were markedly increased in the sub-temporal area.

Additionally, we discovered that patients with iridocyclitis had markedly increased FC signal values between the left V1 and right hippocampus. The hippocampus is a critical subcortical nucleus that is closely associated with in memory function and orientation (56); it is also a component of the Papez circuit, which is involved in memory and emotion (57). Therefore, patients with iridocyclitis might experience dysfunction involving both cognition and memory.

The imaging-mediated diagnosis of iridocyclitis still remains challenging because a confirmed diagnosis is generally made on the basis of clinical symptoms. As a multivariate approach, machine learning has the potential to be more sensitive to spatially distributed and subtle effects in the brain than a standard mass-univariate analysis. To date, no studies have assessed the combined effects of FC and supervised machine learning techniques on iridocyclitis. In the present study, we examined whether differences in FC between individuals with iridocyclitis and HCs could serve as a classification feature to discriminate those groups, based on a machine learning approach using a SVM classifier. Receiver operating characteristic (ROC) analysis were used to evaluate the performance of the classifiers. AUC represents the classification power of the SVM classifier, and a larger AUC indicates better classification ability (58). The AUC of this classification was > 70% when the LOOCV technique was applied (*P*<0.001, non-parametric permutation correction). The LOOCV method uses a single subject from each group as the testing data and the remaining subjects as the training data. The procedure is repeated for each subject; and results from each step are averaged to get a final estimate of classification accuracy (59). These results demonstrated that the SVM approach could reach reliable classification capability with the LOOCV method. In addition, the findings demonstrated that FC methodology could be a promising biological indicator for distinguishing patients with iridocyclitis from HCs.

This study had several limitations. First, its small sample size might have reduced its ability to detect more alterations in neuronal activity; classification power based on the 26 patients with iridocyclitis was still not strong enough. Second, the disease duration and severity of iridocyclitis varied greatly in the included patients, which may also restrict the statistical power of the results. Third, the impacts of physiological noise were not completely eliminated, which may have confounded blood oxygen level-dependent signals. Fourth, although we discovered some changes in FC, the directions of these changes were unknown. Future studies should address these limitations through longitudinal evaluation of a larger dataset and technical improvements in analysis methods. Another independent test sample will also be recruited for testifying classification accuracy in our next work. In addition, multimodal fMRI methods may be conducive to the comprehensive analysis of neuropathological mechanisms in patients with iridocyclitis.

## Conclusion

This study explored iridocyclitis-related cerebral function deficits using an FC approach. The results demonstrated that patients with iridocyclitis exhibited complex disturbances in brain networks, particularly between the V1 and brain regions such as vision-related, somatosensory, and cognition-related regions. These findings may enhance the understanding of the pathological mechanisms of iridocyclitis and indicate that FC signals could be effective biomarkers in the clinical evaluation of neurological impairment in patients with iridocyclitis.

## Data Availability Statement

The original contributions presented in the study are included in the article/supplementary material. Further inquiries can be directed to the corresponding author.

## Ethics Statement

The studies involving human participants were reviewed and approved by Rennin Hospital of Wuhan University. The patients/participants provided their written informed consent to participate in this study.

## Author Contributions

YT contributed to study design, fMRI data analysis, and drafted the manuscript. XH contributed to design the protocol, fMRI data analysis, and data collection. C-XQ contributed to data collection and manuscript discussion. YS conceived the study, reviewed, and revised the manuscript. All authors contributed to the article and approved the submitted version.

## Funding

This research was supported by the National Key R&D Program of China (Grant No. 2017YFE0103400), and by The National Nature Science Foundation of China (Grant No. 81470628).

## Conflict of Interest

The authors declare that the research was conducted in the absence of any commercial or financial relationships that could be construed as a potential conflict of interest.
